# Diversity of microbial communities and soil nutrients in sugarcane rhizosphere soil under water soluble fertilizer

**DOI:** 10.1371/journal.pone.0245626

**Published:** 2021-01-22

**Authors:** Huan Niu, Ziqin Pang, Nyumah Fallah, Yongmei Zhou, Caifang Zhang, Chaohua Hu, Wenxiong Lin, Zhaonian Yuan

**Affiliations:** 1 College of Agricultural, Fujian Agriculture and Forestry University, Fuzhou, China; 2 Key Laboratory of Sugarcane Biology and Genetic Breeding, Ministry of Agriculture, Fujian Agriculture and Forestry University, Fuzhou, China; 3 Province and Ministry Co-sponsored Collaborative Innovation Center of Sugar Industry, Guangxi University, Nanning, China; University of Florida, UNITED STATES

## Abstract

The dynamics of soil microbial communities are important for plant health and productivity. Soil microbial communities respond differently to fertilization. Organic water soluble fertilizer is an effective soil improver, which can effectively improve soil nutrient status and adjust soil pH value. However, little is known about the effects of water soluble fertilizers on soil microbial community, and the combined effects on soil nutrients and sugarcane productivity. Therefore, this study sought to assess the effects of water soluble fertilizer (1,050 kg/hm^2^ (WS1), 1,650 kg/hm^2^ (WS2)) and mineral fertilizer (1,500 kg/hm^2^ (CK)) on the soil microbial community, soil nutrients and crop yield of sugarcane. The results showed that compared with CK, the application of water soluble fertilizers (WS1 and WS2) alleviated soil acidity, increased the OM, DOC, and AK contents in the soil, and further improved agronomic parameters and sugarcane yield. Both WS1 and WS2 treatments significantly increased the species richness of microorganisms, especially the enrichment of beneficial symbiotic bacteria such as Acidobacteria and Planctomycetes, which are more conducive to the healthy growth of plants. Furthermore, we found that soil nutrient contents were associated with soil microbial enrichment. These results indicate that water soluble fertilizer affects the enrichment of microorganisms by improving the nutrient content of the soil, thereby affecting the growth and yield of sugarcane. These findings therefore suggest that the utilization of water soluble fertilizer is an effective agriculture approach to improve soil fertility.

## 1. Introduction

As the main sugar crop, sugarcane has become an important pillar of economic development and the economic source of people’s income growth in many regions. In China, sugarcane cultivation is mainly practiced in Yunnan, Guangxi, and other regions. However, due to the poor and acid soil in Guangxi, Yunnan, and other areas, the nutrient content of the soil cannot meet the physiological needs of sugarcane growth, so a large amount of fertilization is needed to enhance the sugarcane yield [1]. In 1840, the famous German chemist Libische put forward the theory of plant mineral nutrition. In 1842, the British Louth obtained the patent right of calcium persulfate made from bone meal and sulfuric acid, thus creating a 160 year history of fertilizer use [2]. Fertilizer can quickly and effectively provide essential nutrients for plants, and at the same time, it can improve soil properties, improve soil fertility and promote plant growth [3]. Fertilizer has been playing an important role in agricultural production, and with the development of inorganic fertilizer use is also increasing. Although the use of chemical fertilizer improves crop yield, it also brings a lot of environmental pollution problems. For instance, Zhang X et al. reported that the excess use of chemical fertilizer causes excessive nitrate in soil, contaminates of ground water [4]. Moreover, Guo et al. documented that the long term accumulation of mineral fertilizer in soil leads to metal toxicity, diminishes nutrient use efficiency, causes soil acidification, which in term negatively affects crop growth, and productivity [5].

Sugarcane fertilization is greatly affected by the external environmental factors, with obvious regional differences. So far, the experimental research on sugarcane fertilization in China is still in the primary stage, and there is no consistent conclusion on the effect of fertilization. Different fertilizer and fertilization levels will have a great impact on soil microbial diversity and chemical properties [6]. Soil organic matter is one of the indicators of soil fertility, and the more abundant soil microorganisms, the higher the content of soil organic matter. The activity of soil microorganisms can truly reflect the change and distribution of soil nutrients [7]. The application of organic fertilizer can significantly improve the soil microbial environment, increase the number of soil microbial groups, cause the change of soil carbon storage, and then affect the carbon sink of the terrestrial ecosystem. Water soluble fertilizer is a typical high-efficiency organic fertilizer, which can not only effectively reduce the degree of soil acidification, but also improve the status of soil nutrients and crop productivity [8]. However, as a result of limited detection methods and the complexity of microbial metabolic pathways, information on microbial diversity under water soluble fertilizer in sugarcane cultivation field is scared.

Based on the continuous development of bioinformatics, high-throughput sequencing data [9,10], fuguild, picrust, and other tools have been applied to the diversity of bacteria [11,12]. In view of this, these bioinformatics tools have been used to predict soil microorganisms diversity in response to the utilization of water soluble fertilizer in farmland soil, so as to provide the latest understanding and reference for future research.

In this study, high-throughput sequencing was employed to assess the effect of water soluble fertilizer application on soil microbial community composition response under sugarcane planting system. In addition, we studied the effect of water soluble fertilizer on soil characteristics, microbial community structure, and its overall impact on crop productivity. In general, the purpose of current research is to use multidisciplinary strategies to evaluate the impact of water soluble fertilizer on the composition of soil microbial communities. Assuming that the application of water soluble fertilizer will affect the soil acidification and nutrition, we believe that (a) water soluble fertilizer has a great impact on the soil characteristics, resulting in changes in soil microbial structure and diversity; (b) Changes in soil physical and chemical properties can reflect changes in microbial community composition. Moreover, we hypothesized that (c) water soluble fertilizers affect microbial population, which in turn leads to changes in the number of harmful microorganisms, thus affecting the sustainability and performance of agricultural ecosystems.

## 2. Materials and methods

### 2.1. Experimental design and sample collection

Field experiment was conducted in Chongzuo city, Guangxi province, China (north latitude 22°49’, longitude 107°76’), with annual average temperature and rainfall of 21.7°C and 1,121 mm respectively. Before the cultivation of sugarcane, the physical and chemical properties of the soil were determined: pH value was (4.18), soil organic matter (19.87 g/kg), total nitrogen (1.45 g/kg), total phosphorus (1.23 g/kg), and total potassium (1.21 g/kg). Fertilizer (conventional compound fertilizer N:P:K = 15:15:15) and water soluble fertilizer (containing water-soluble organic matter ≥220 g/L, nitrogen, phosphorus and potassium ≥250 g/L, biochemical fulvic acid = 220 g/L, amino acid = 220 g/L, zinc, boron, copper, iron, manganese, molybdenum ≥4000 ppm, and water insoluble matter ≤50 g/L) were used as comparison fertilizers. Three treatments were set up in total based on sugarcane crop recommendations: (1) CK, fertilizer 1,500 kg/hm^2^ [13,14] (2) WS1, water soluble fertilizer 1,050 kg/hm^2^ (3) WS2, and water soluble fertilizer 1,650 kg/hm^2^. The experiment was laid out in a randomized block design, replicated thrice. Each plot consisted of 5 rows, each measured 50 m, with 1.3 m row spacing. The experiment covered an area of 325 m^2^. The fertilizer was applied at two different times: April 2019 (40% organic fertilizer and chemical fertilizer) and June 2019 (60% organic fertilizer and chemical fertilizer).

Data collection of plants parameters began on November 28, 2019, followed by the collection of soil samples on the same day. For each plot, soil samples were randomly collected from six spots (2.5 cm in diameter and 0 to 20 cm in depth), homogenized, and mixed appropriately forming one sample. Fresh soil samples are sieved using a 2 mm sieve to remove non-soil components (e.g. plant and animal remains, rocks, etc.). The fresh soil samples were divided into three parts for preservation: one part of the soil was preserved at -80°C, the other part was air-dried for physical and chemical properties determination, and the last part was temporarily preserved at 4°C for soil microbial determination and DNA extraction.

### 2.2. Measurement of sucrose content and theoretical yield

In each row, thirty sugarcane plants were randomly selected, and each plant height and plant diameter were measured in centimeters (cm) using a tape and a vernier caliper. To determine sucrose content, an Extech Portable Sucrose Brix Refractometer (Mid-State Instruments, San Luis Obispo, CA, USA) was used, which was measured using the formula: sucrose (%) = Brix (%) × 1.0825 − 7.703 [15].The theoretical sugarcane production was estimated using the following equations [15]:

- Single stalk weight (kg) = (stalk diameter (cm))^2^ × (stalk height (cm) − 30) × 1 (g/cm^3^) × 0.7854/1000.
- Theoretical production (kg/hm^2^) = single stalk weight (kg) × productive stem numbers (hm^2^).

### 2.3. Measurement of soil chemical properties

Soil suspension with water (1:2.5 W/V) was prepared in order to measure soil pH using a pH meter (PHS-3C, INESA Scientific Instrument Co, Ltd, Shanghai, China) [16]. (OM) soil organic matter was determined by the Walkley-black method, which was based on the oxidation of soil organic matter by K_2_Cr_2_O_7_ and H_2_SO_4_, and then titrated with FeSO_4_ [17]. Dissolved organic carbon content (DOC) was calculated using redox titration with 0.8 mol/L K_2_Cr_2_O_7_ [18]. Soil available phosphorus (extracted by Bray No. 1) was determined according to Bray and Kurtz (1945) and Menage and Pridmore (1973) approach [19]. Both soil available nitrogen (AN) and available potassium (AK) were determined us alkaline hydrolyzable diffusion method [20], and flame photometry method [21], respectively. Descriptive statistical analyses were performed using MS Excel 2013, DPS software 12.26.

### 2.4. Soil DNA extraction, PCR amplification and sequencing

Power Soil DNA Isolation Kit (MoBio Laboratories Inc., Carlsbad, USA) was used to extract soil DNA from each soil sample according to the manufacturer’s instructions. A NanoDrop 2000 spectrophotometer (Thermo Scientific, Waltham, Massachusetts, USA) was used to assess the quality and concentration of soil DNA. A sequencing library was prepared using primers sets 27F/1492R [22,23] and ITS9munngs/ITS4ngsUni [24] to amplify the 16S rRNA gene and ITS. PCR conditions were 95°C for 5 min, followed by 30 cycles of 95°C for 30 s, 55°C for 30 s, and 72°C for 1min 30 s, with a final extension at 72°C for 7 min (GeneAmp 9700, ABI, California CA, USA). According to the electrophoresis results, quantification was done using ImageJ software. After quantification, samples are mixed according to the required output data volume and fragment size of each sample. And 0.8X magnetic beads (MagicPure Size Selection DNA Beads) was used to recover and purify. The SMRTbell-adapted libraries were sequenced on a SMRT cell, carried out by P6C4v2 chemistry [25,26] chemical reagents to run on the Pacific Biosciences (PacBio) RS II platform [27,28].

### 2.5. Processing and analyzing of sequencing data

Output files were processed and assembled into CCS reads using CCS2 v3.0.1 setting the minimum passes to 3 [29], minimum signal-to-noise ratio (SNR) to 4, minimum length to 1200, minimum predicted accuracy to 0.9, and the minimum Z-score to −5. Consensus sequences longer than 1600 bp were removed. Usearch (version 8.1) [30] tool was employed to analyze the result. Using the UCLUST denovo method, the sequences were clustered into operational taxa with 97% similarity (OTU) to generate sparse curves and abundance and diversity indices were calculated [31]. Both Alpha diversity and species richness of the single sample were also examined [32]. Mothur (Version V. 1.30) software was employed to determine Alpha diversity index of the sample. Beta diversity analysis was performed using QIIME software to compare the similarities of different samples for species diversity. Principal Coordinates Analysis (PCoA) [33] PCoA was used for the classification of samples, which further demonstrate the differences in species diversity among samples, and R (version 3.2.2) tool was to draw the PCoA analysis chart respectively [34]. The circlize package in the R (version 3.2.2) was used to draw chord diagrams [35], and ggplot2, magrittr and ggpubr packages were used to visually analyze the species diversity [36]. In order to understand the microbial differences among WS1, WS2 and CK, DESeq2 package in the R (version 3.2.2) was adopted to investigate the different species of 16S and ITS at the OTUs level, and then used the Manhattan display according to Zhang et al. [37] methods. A Pearson’s correlation analysis was calculated to determine the interaction between soil properties and microbial taxa (phylum), using R-software [38]. In order to assess the relationship between environmental factors and microbial community structure, a Mantel test was performed [39]. Redundancy analysis (RDA) was then carried out further examine the influence of soil physiochemical properties on bacteria and fungi phylum abundance using R (version 3.2.2) [40].

## 3. Results

### 3.1. Sugarcane agronomic properties and production

Sugarcane Agronomic Properties results showed that the water soluble fertilizer had an effect on sugarcane growth parameters (Table 1). Compared with the control, the application of water-soluble fertilizers (WS1 and WS2) in sugarcane field increased the sucrose content, growth parameters (available stalk number, stalk height, and weight) and theoretical yield of sugarcane, but there was no significant difference in statistical level. These results indicated that the sugarcane planting system based on water-soluble fertilizer may affect a certain index of sugarcane growth parameters, but has no significant effect on the overall growth characteristics of sugarcane.

**Table 1:** Impact of water-soluble application on sucrose content, growth parameters, and yield of sugarcane.

Treatment	Sucrose content (%)	Available stalk number (h/m^2^)	Stalk height (cm)	Stalk diameter (cm)	Single stalk weight (kg)	Theoretical production (kg/hm^2^)
CK	13.04±1.21^a^	43,590±3775^ab^	269.1±8.6^a^	3.17±0.05^a^	1.90±0.04^a^	82,537±5326^b^
WS1	13.83±0.84^a^	42,863±3329^b^	275.5±6.0^a^	3.18±0.33^a^	1.97±0.43^a^	84,490±9421^ab^
WS2	13.20±0.84^a^	50,128±5766^a^	275.8±3.1^a^	3.18±0.10^a^	1.96±0.15^a^	98,533±7542^a^

For every treatment with replicates, one-way variance analysis (ANOVA) by LSD test (p < 0.05) was performed. Different letters indicate a significant difference among treatments. CK: N, P, and K fertilizer; WS1: water soluble fertilizer 1,050 kg/hm^2^; WS2: water soluble fertilizer 1,650 kg/hm^2^.

### 3.2. Soil physiochemical properties

**Fig 1:**
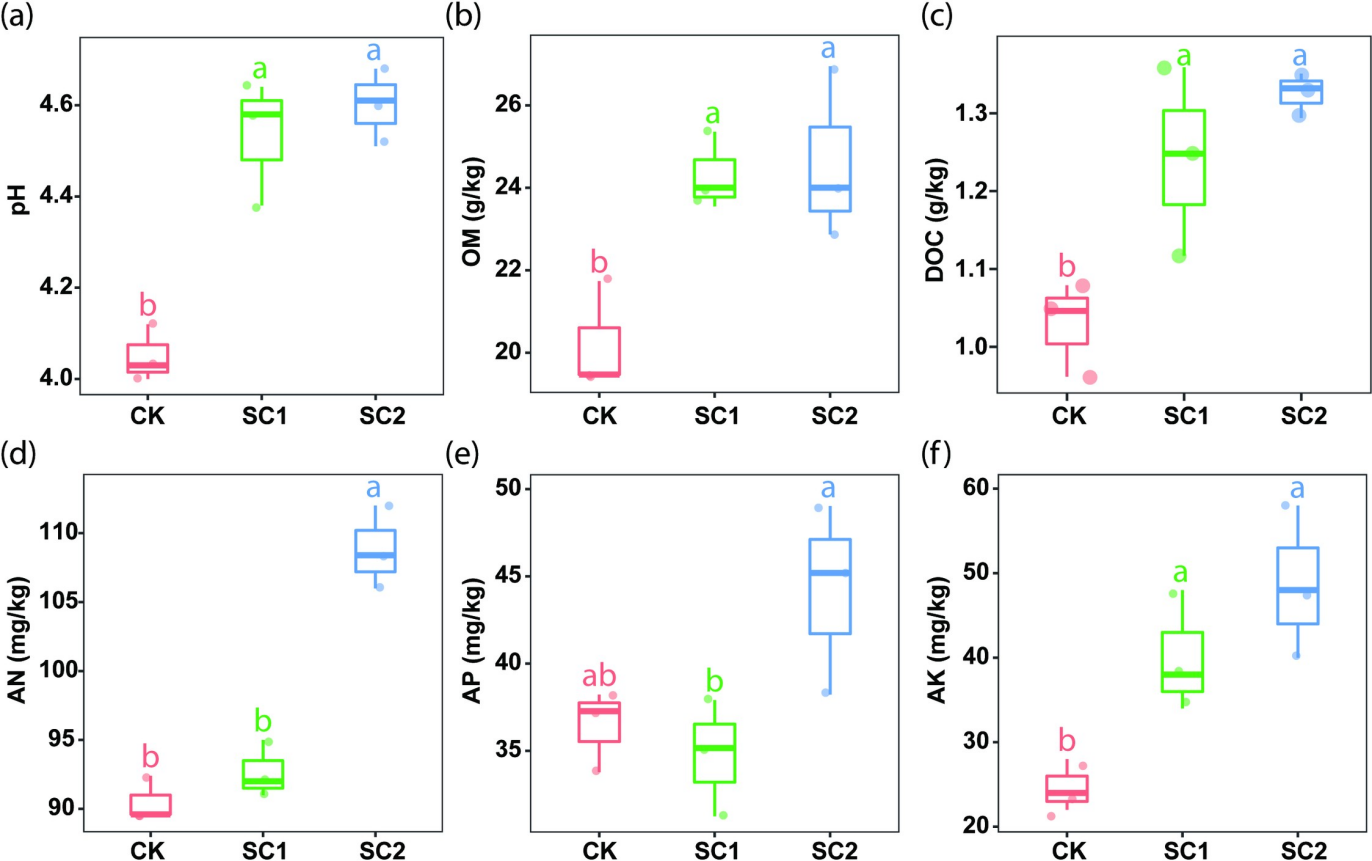
Box plot indicating the effect of water-soluble fertilizer application on soil physiochemical properties under sugarcane cultivation. Lowercase letters indicate the difference among treatments based on the LSD test (p < 0.05). AN, available nitrogen; AP, available phosphorus; AK, available potassium; OM, organic matter; DOC, dissolved organic carbon; CK: N, P, and K fertilizer; WS1: water soluble fertilizer 1,050 kg/hm^2^; WS2: water soluble fertilizer 1,650 kg/hm^2^.

Fig 1 was obtained by measuring the physicochemical properties of the soil gathered from control (CK) and water soluble fertilizer treatment (WS1 and WS2) treatments. Compared with CK, WS1 and WS2 treatments effectively diminished soil acidity and increased the contents of OM, DOC, and AK in the soil. While, the content of AN in soil increased significantly only in WS2 treatment. These results show that a sugarcane cropping system based on water-soluble fertilizer application can efficiently alleviates soil acidification and improves soil fertility.

### 3.3. Microbial beta diversity

**Fig 2:**
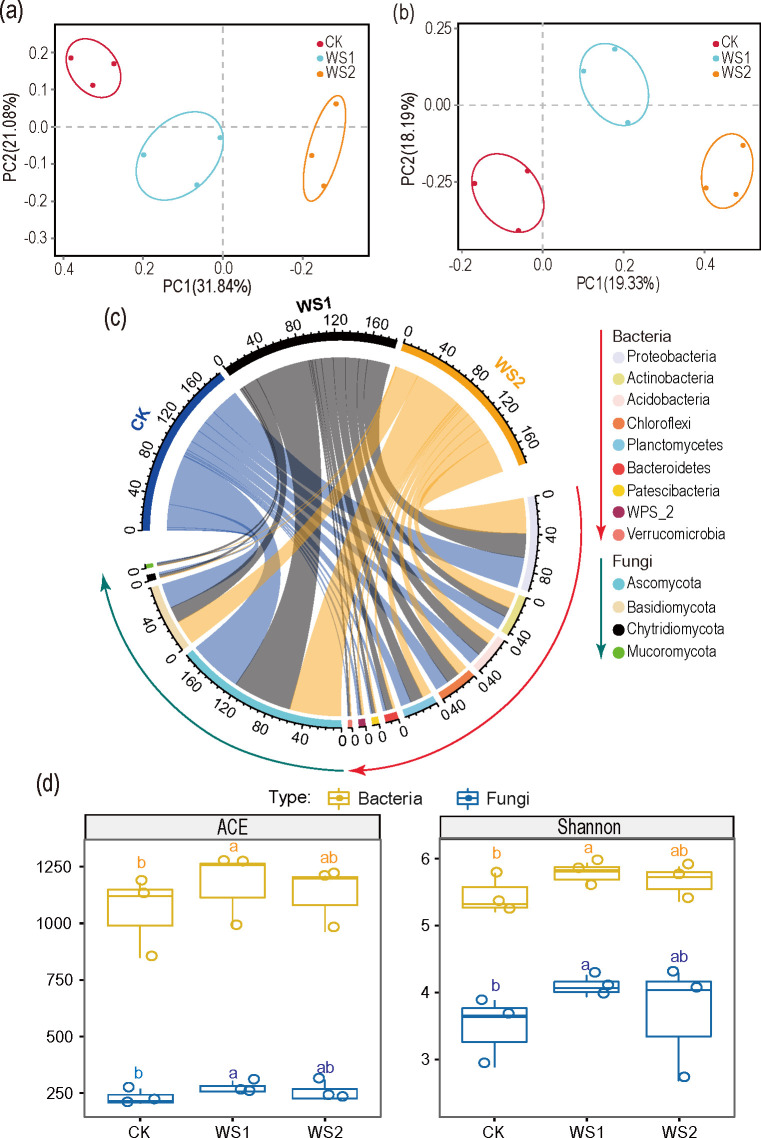
Microbial diversity analysis. Analysis of principal coordinates (PCoA) of bacterial (a) and fungal communities (b) of the Bray–Curtis in the various treatments were assessed. The Circos diagram depicts the top microbial composition at the phyla level in three sugarcane fields (c). Values means (n = 3). Box plots represent alpha diversity indices, including microbial community richness (ACE) and diversity (Shannon) under different treatments (d). Lowercase letters indicate the difference among treatments based on the LSD test (p < 0.05). CK: N, P, and K fertilizer; WS1: water soluble fertilizer 1,050 kg/hm^2^; WS2: water soluble fertilizer 1,650 kg/hm^2^.

The principal axis analysis (PCoA) based on the Bray-Cutis distance indicated that the fungal and bacterial communities in the rhizosphere soil of sugarcane under different fertilizer treatments were separated on the first axis (P<0.001, PERMANOVA used the Adonis function replacement test). This includes 37.52% of fungi and 52.92% of bacteria. The analysis further confirmed that the microbial group patterns in WS1 and WS2 were separated from CK (Fig 2C and 2D), The results demonstrated that water soluble fertilizer significantly changed the composition of soil microbial community.

### 3.4. Microbial alpha diversity

Microbial diversity and richness of various samples were measured, and significant differences were observed among CK and WS1 treatments. The curve reveals a smooth trend (S1 Fig), which confirms that sequencing depth is suitable for determining soil microbial diversity and richness in all treatments. Compared with CK, WS1 treatment significantly enhanced microbial community richness under sugarcane cultivation, and the alpha diversity of microorganisms (Shannon diversity index) also showed the same trend (Fig 2D), thus indicating that the utilization of water soluble fertilizer can improve the species of bacteria and fungi in sugarcane field.

### 3.5. Microbial community composition

**Fig 3:**
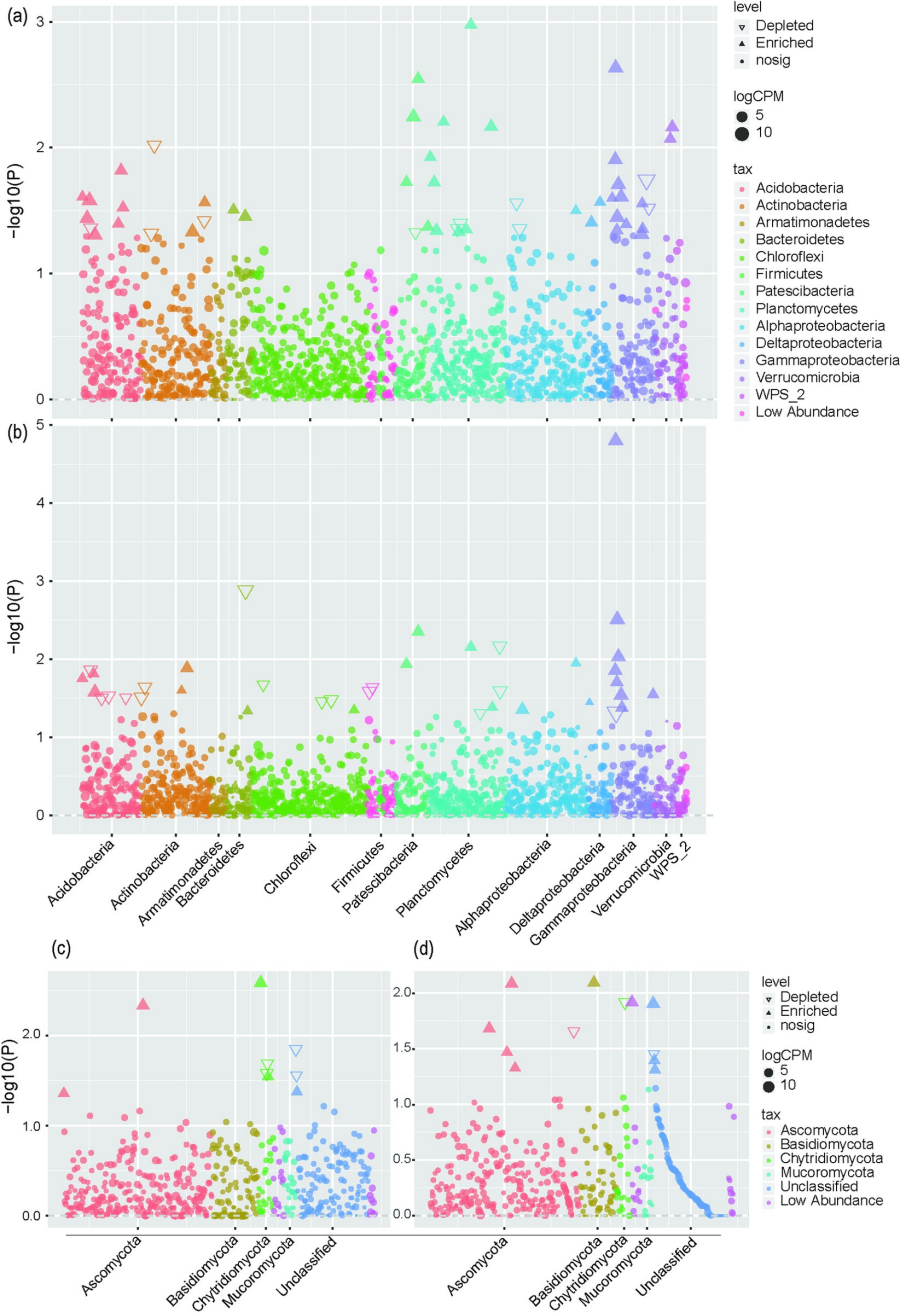
Taxonomic diagram of different bacterial and fungal between the WS1 and WS2 soil microbiota. Manhattan plot showing OTUs enriched in soil in WS1 (a, c) and WS2 (b, d). CK: N, P, and K fertilizer; WS1: water soluble fertilizer 1,050 kg/hm^2^; WS2: water soluble fertilizer 1,650 kg/hm^2^.

In all the treatments, the most dominant bacterial phyla were identified, phylum Proteobacteria, Actinobacteria, Chloroflexi, Acidobacteria, Planctomycetes, Bacteroidetes, WPS-2, and Patescibacteria were highly dominate (Fig 2C). Next, we studied the differences of microbial communities in sugarcane roots under three treatments at OTUs level. We analyzed the enrichment of OTUs according to their taxonomy using Manhattan plots (Fig 3). Among various treatments, Acidobacteria and Planus, OTUs were significantly enriched in WS1 treatment, compared with CK treatment (Fig 3A). Whereas in WS2 treatment, Protein bacteria enriched OTUs were significantly higher compared to CK treatment (Fig 3B). The abundant fungal phyla, such as Ascomycota, Basidiomycota, and Chytridiomycota, were observed in all the treatments. However, the OTUs of Chytridiomycota in WS1 and WS2 treatments decreased relative to that under CK treatment (Fig 3C and 3D). The OTUs of Ascomycota and Cryptomycota in WS1 were higher than that of CK, while the OTUs of Burkholderia-Caballeronia-Paraburkholderia in WS1 decreased, however in WS2 treatment Burkholderia-Caballeronia-Paraburkholderia enriched OTUs were higher than that of CK treatment.

### 3.6. Correlation among soil chemical properties and microbial community

**Fig 4:**
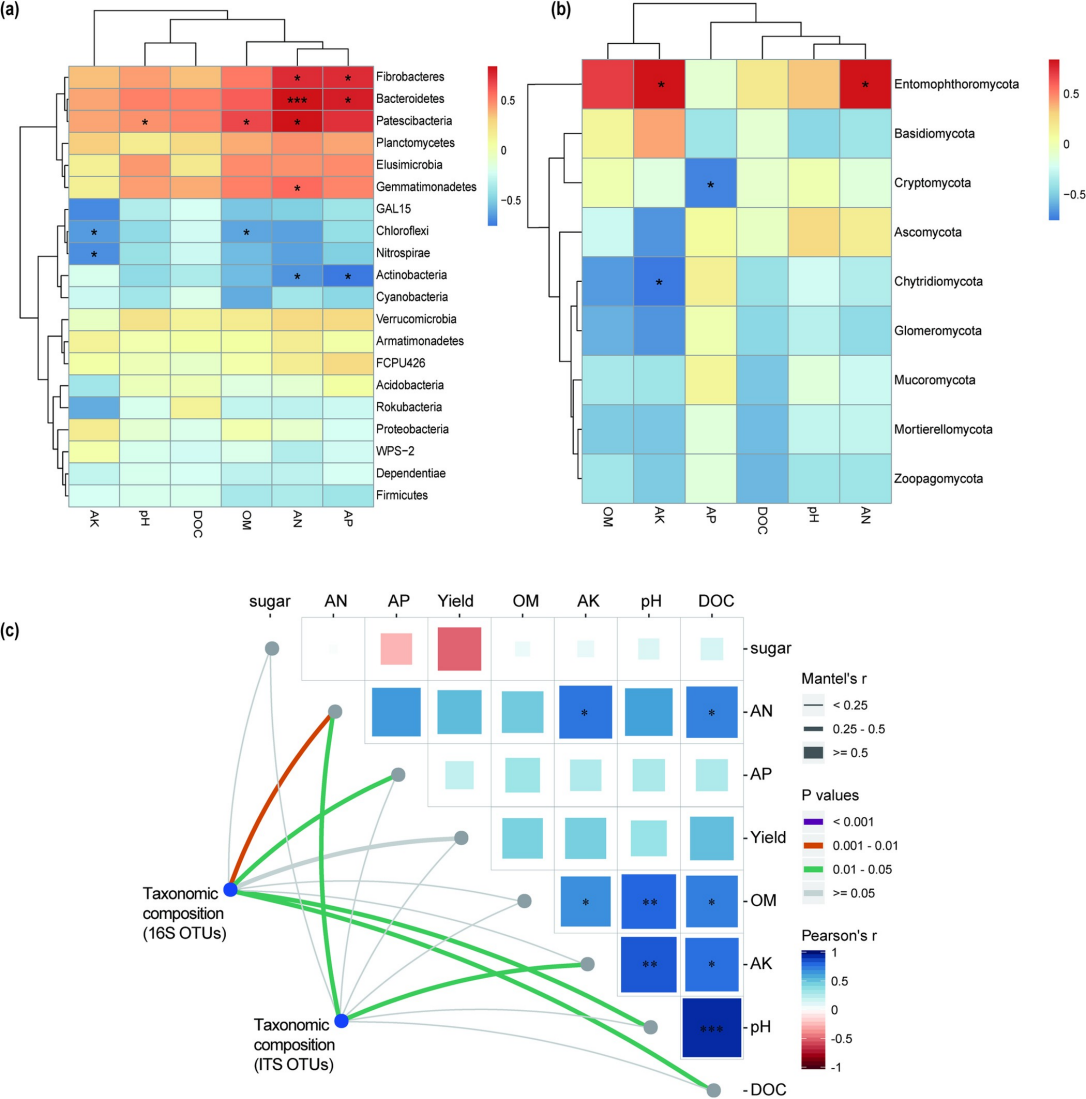
Correlation analysis between soil chemical properties and microbial community. Correlation between soil physiochemical properties and bacterial phyla (a), and fungus phyla (b) levels. Note: asterisk mark represents the level of significance. *0.01<p<0.05, **0.001<p<0.01, ***p<0.001 (c) Pairwise comparisons of environmental factors are shown with a color gradient denoting Pearson’s correlation coefficients. Taxonomic [based on two independent methods: 16S OTUs, ITS OTUs] community composition was associated with each environmental factor by partial Mantel tests. Edge width corresponds to the Mantel’s r statistic for the corresponding distance correlations.

Both distance-corrected dissimilarities of taxonomic and community composition were correlated with environmental factors (Fig 4C). Distance correction differences in classification and community composition were correlated with differences in environmental factors. The analysis revealed that DOC content in soil had the strongest correlation with soil pH, while the correlation with yield was not significant. Except for sugarcane yield and phosphorus content, the other nutrients showed positive correlation. Nitrogen content had a significant effect on microbial diversity, and soil pH value and DOC content also had a significant effect on bacterial diversity (Fig 4C). In terms of the diversity of bacteria, nitrogen content was significantly correlated with Fibrobacteres, Bacteroidetes, Patescibacteria, Gemmatimonadetes and Actinobacteria, while soil phosphorus content was also significantly correlated with Fibrobacteres, Bacteroidetes and Actinobacteria (Fig 4A). Soil physiochemical properties had little influence on fungal diversity, in which the soil available potassium was significantly related to the Entomophthoromycota and Chytridiomycota. Furthermore, soil available nitrogen was significantly associated with phylum Entomophthoromycota, while soil available phosphorus had a negative correlation with Cryptomycota (Fig 4B).

### 3.7. Redundancy analysis

**Fig 5:**
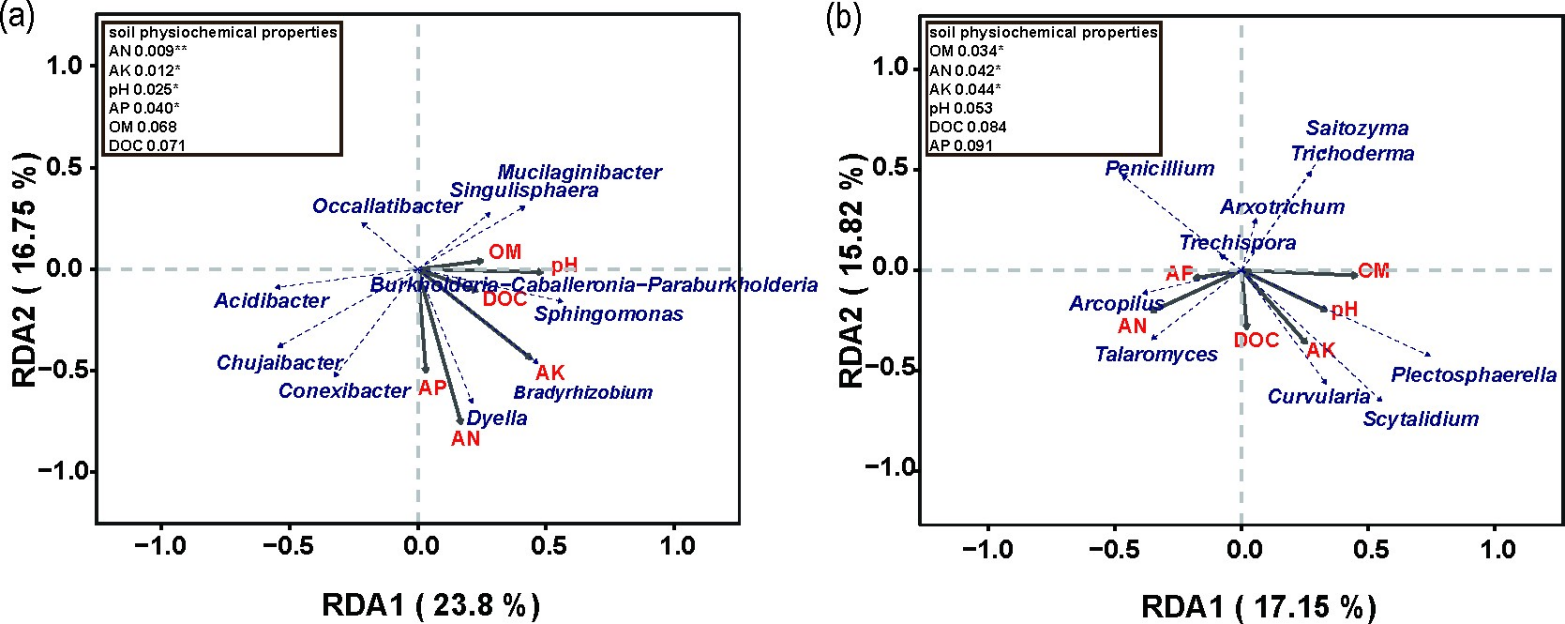
Redundancy analysis (RDA) indicating shift in bacterial (a) and fungal (b) communities caused by soil physiochemical properties. AN, available nitrogen; AP, available phosphorus; AK, available potassium; OM, organic matter; DOC, Dissolved organic carbon; In the top-left, the soil properties were fitted to the ordination plots using 999 permutations test (P-values) *0.01<p<0.05,**0.001<p<0.01; CK: N, P, and K fertilizer; WS1: water soluble fertilizer 1,050 kg/hm^2^; WS2: water soluble fertilizer 1,650 kg/hm^2^.

Redundancy analysis (RDA) and Pearson correlation analysis were used to further determine the response of microbial structure to environmental factors. RDA results showed that pH, AN, AP, AK, OM and DOC accounted for 40.55% and 32.97% of the total migration of bacterial and fungal communities, respectively (Fig 5A and 5B). Burkholderia−Caballeronia−Paraburkholderia was positively correlated with pH, OM, and DOC, while Dyella and Bradyrhizobium were positively correlated with AN, AP, and AK. On the other hand, Acidibacter was negatively correlated with pH and OM, and Occallatibacter was negatively correlated with AK (Fig 5A). Plectosphaerella showed positive association with soil pH and negatively correlated with soil AP. Moreover, there was a positive correlation between Arcopilus and AP and a negative correlation between Arcopilus and OM (Fig 5B). RDA double point method and further replacement test showed that soil AN AK, pH and AP contents were a key predictor of bacterial community structure. Meanwhile, soil OM, AN and AK were the main influencing factors shifting fungal community structure.

## 4. Discussion

As expected, WS treatment significantly increased the sugarcane yield compared with CK, which might be mainly attributed to soil fertility and the effect of the functional strains in the WS. The sugar yield was closely associated with the sugarcane yield and sucrose content, which were the final products of sugarcane processing. However, no significant difference in the mean sucrose content was observed in the soil type of WS in our study. In addition, water soluble fertilizer reduced soil acidification, and improved soil nutrient status, especially OM, DOC, and AK in a sugarcane cropping system [41–44]. Hence, we can infer from this study that alleviating soil acidification and improving soil nutrient status in water soluble fertilizer treated fields (Fig 1) may help improve agronomic parameters and sugarcane production [45–47]. Compared with CK, soil amended with water soluble fertilizer improved soil quality thereby by stimulating the growth of soil microorganisms. Our study showed that the soil microbial richness in WS1 and WS2 treatments was significantly higher relative to that in CK treatment, which further proved that the application of water soluble fertilizer may respond to the increase of nutrients, thus stimulating the soil microbial population and leading to the change of species richness. The positive effects of water soluble fertilizers on microbial biomass have been well documented in other agricultural systems [48]. In summary, the application of water soluble fertilizers improves productivity by reducing soil acidification, increasing the availability of nutrients (OM, DOC, and AK), which in term increased soil microbial biomass. We observed an increase in the agronomic parameters and yield of sugarcane in the soil treated with water soluble fertilizers compared to the control group.

Soil microbiota plays an essential role in soil function and sustainable development of ecosystem [49,50]. Therefore, studying the response of soil microbial diversity to the application of water soluble fertilizer is pivotal. Changes in microbial diversity is considered to be the most important indicators for ecosystem service function to restore or destroy soil function (promote plant growth or inhibit disease) [51]. Therefore, the application of water soluble fertilizer is conducive for the restoration of soil function, thus increasing sugarcane yield. Water soluble fertilizer application in sugarcane cropping systems significantly altered the soil microbial community (Fig 2A–2D). Soil chemistry plays an important role in microbial communities [52,53]. RDA and Pearson correlation results (Fig 5) revealed a significant structural changes in soil microbial communities, which can be attributed to the changes in soil chemical properties [53]. In this study, the composition of bacterial and fungal communities were significantly correlated with the application of water soluble organic fertilizer.

Compared with CK treatment, the Acidobacteria, Planctomycetes, Proteobacteria in soils treated with water-soluble fertilizer were more abundant (Fig 2C). These bacteria are associated with soil acid bacteria influenced at higher soil pH, and can significantly promote carbon cycling [54] and eliminate wilt [55]. Planctomycetes is an Anammox bacterium that plays a significant role in the nitrogen and carbon cycle [56]. The application of high levels of water soluble fertilizer increased the abundance of the Burkholderia-Caballeronia-Paraburkholderia. Previous findings documented that Burkholderia sp. strain PsJN, which was initially designated as Pseudomonas sp. strain PsJN [57], is an effective growth-promoter of plant that was isolated as a contaminant from Glomus vesiculiferum-infected onion roots. This bacterium is known to enhance the growth of potatoes crops [57], vegetables, and grapevines [58] via reduction of the level of the inhibitory hormone ethylene by a high level of 1-aminocyclopropane-1-carboxylic acid (ACC) deaminase that is secreted. Previous studies have shown that inorganic fertilization, especially nitrogen [59], is the main reason for the low abundance of ascomycetes, which may be one of the reasons for the aggravation of sugarcane disease after applying inorganic fertilizer. These results suggest that water soluble fertilizer has a positive effect on sugarcane growth by stimulating beneficial microbial communities and microbial functions associated with soil and plants.

## 5. Conclusions

In summary, we used high-throughput sequencing technology to monitor the diversity of the soil microbial community in the rhizosphere of sugarcane under water-soluble fertilizer treatment for the first time, and analyzed the relationship between the physical and chemical properties of the soil and the microbial community. Finally, it was discovered that water soluble fertilizer application reduced soil acidity, improved the microbial flora, and enhanced the abundance of soil microbial species. These findings therefore suggest that the utilization of water soluble fertilizer is an effective agriculture approach to improve soil fertility.

## Supporting information

S1 Fig Rarefaction curves at an OTU threshold of 97% sequence similarity (a, b) for soil samples taken derived from three sugarcane fields.
(TIF)
