# Supplementary figures and images for: Diversity of microbial communities and soil nutrients in sugarcane rhizosphere soil under water soluble fertilizer
Source: PLoS One. 2021 Jan 22;16(1):e0245626. doi: 10.1371/journal.pone.0245626 (PMC7822549; doi:10.1371/journal.pone.0245626)

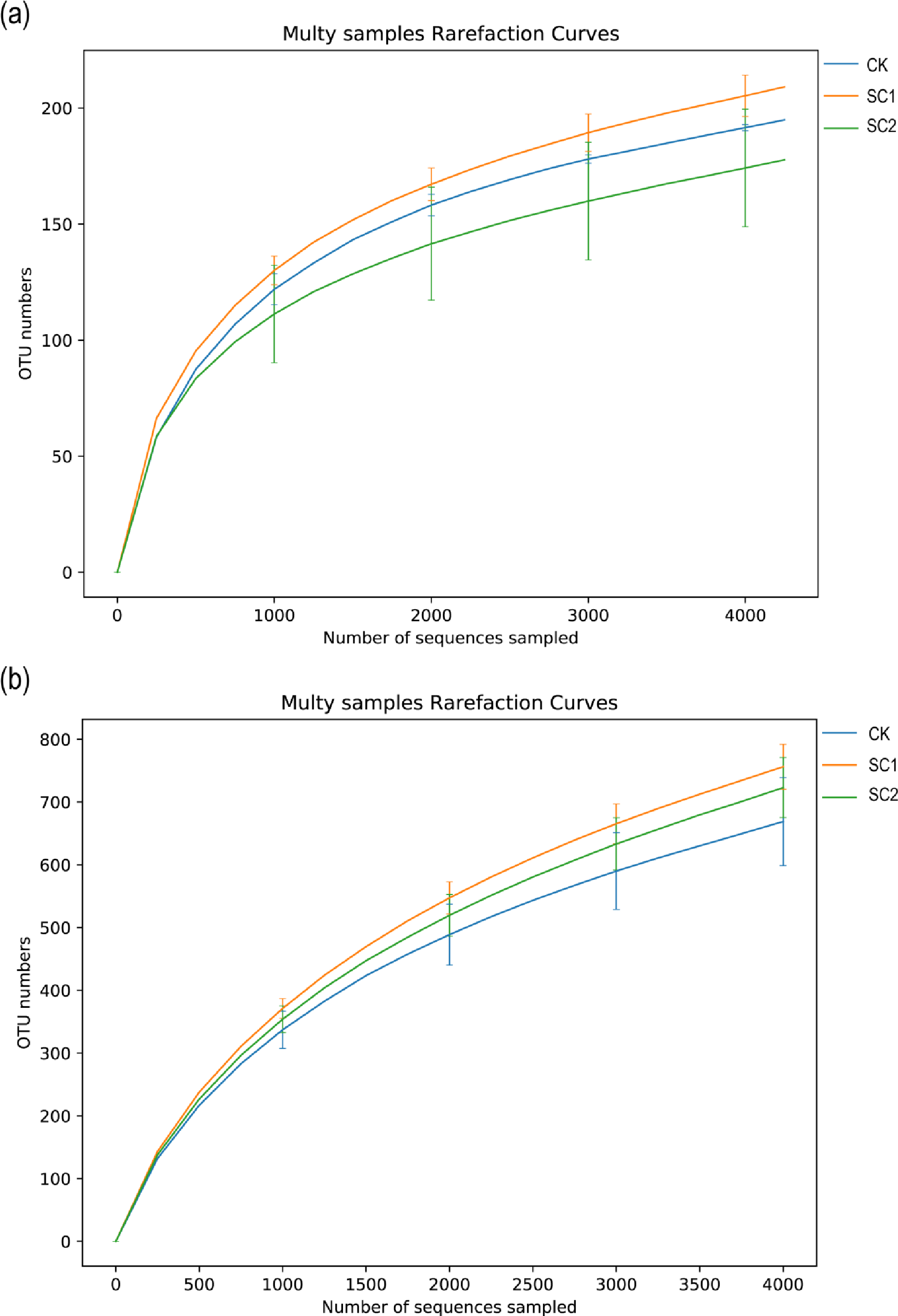

Supplement: S1 Fig — Rarefaction curves at an OTU threshold of 97% sequence similarity (a, b) for soil samples taken derived from three sugarcane fields. (TIF) [file pone.0245626.s001.tif]
